# The non-coding genome in genetic brain disorders: new targets for therapy?

**DOI:** 10.1042/EBC20200121

**Published:** 2021-10-27

**Authors:** Eva Medico-Salsench, Faidra Karkala, Kristina Lanko, Tahsin Stefan Barakat

**Affiliations:** 1 Department of Clinical Genetics, Erasmus MC University Medical Center, Rotterdam, The Netherlands; 2 Department of Neuroscience, Erasmus MC University Medical Center, Rotterdam, The Netherlands

**Keywords:** clinical genetics, epigenomics, functional genomics, gene expression and regulation, Non-coding regulatory elements, therapy

## Abstract

The non-coding genome, consisting of more than 98% of all genetic information in humans and once judged as ‘Junk DNA’, is increasingly moving into the spotlight in the field of human genetics. Non-coding regulatory elements (NCREs) are crucial to ensure correct spatio-temporal gene expression. Technological advancements have allowed to identify NCREs on a large scale, and mechanistic studies have helped to understand the biological mechanisms underlying their function. It is increasingly becoming clear that genetic alterations of NCREs can cause genetic disorders, including brain diseases. In this review, we concisely discuss mechanisms of gene regulation and how to investigate them, and give examples of non-coding alterations of NCREs that give rise to human brain disorders. The cross-talk between basic and clinical studies enhances the understanding of normal and pathological function of NCREs, allowing better interpretation of already existing and novel data. Improved functional annotation of NCREs will not only benefit diagnostics for patients, but might also lead to novel areas of investigations for targeted therapies, applicable to a wide panel of genetic disorders. The intrinsic complexity and precision of the gene regulation process can be turned to the advantage of highly specific treatments. We further discuss this exciting new field of ‘enhancer therapy’ based on recent examples.

## Introduction

The human genome contains more than 20,000 protein-coding genes whose expression needs to be precisely regulated in order for normal development and physiology to occur. A key component of this well-tuned orchestra of gene regulation are non-coding regulatory elements (NCREs), which modulate the magnitude, timing and cell specificity of gene expression. The cross-talk between coding and non-coding genome relies on multiple factors, from DNA sequence to 3D chromatin structure. As one of the most complex organs of the human body, the brain is particularly dependent on the correct timing, location and level of gene expression, enabling its development and functioning during life. It is therefore not surprising, that alterations in the regulatory processes of gene regulation, including chromatin modifications, often lead to neurodevelopmental disorders [1–6]. Constantly improving genetic diagnostic techniques, based on next-generation sequencing (NGS), are currently the most effective in identifying disease-causing (e.g. pathogenic) variants in the coding sequences [7]. However, in more than 50% of individuals affected by neurodevelopmental disorders a genetic cause remains elusive despite a high clinical suspicion for a genetic disorder, and it seems plausible that the explanation for this ‘missing heritability’ can be found in the non-coding genome in a significant number of cases [7]. The limited functional annotation of non-coding sequences and limitations in our understanding of NCRE function impedes and perplexes the interpretation of non-coding variants in a diagnostic setting. This, however, is anticipated to change as we witness more studies bridging the fundamental and clinical aspects of NCREs. Also, more than 90% of disease-associated single nucleotide polymorphisms (SNPs) identified by genome-wide association studies (GWASs) are located in non-coding regions of the genome [8], reinforcing the need to better understand mechanisms of gene regulation, NCREs and the non-coding genome.

In this review, we first discuss the basics of gene regulatory mechanisms and methods that can help to functionally annotate NCREs. We then highlight a number of examples where alterations of NCREs lead to disease and discuss potential options to modify NCREs as future treatment modalities for genetic disorders.

### Mechanisms of gene regulation by the non-coding genome in a nutshell

**Figure 1:**
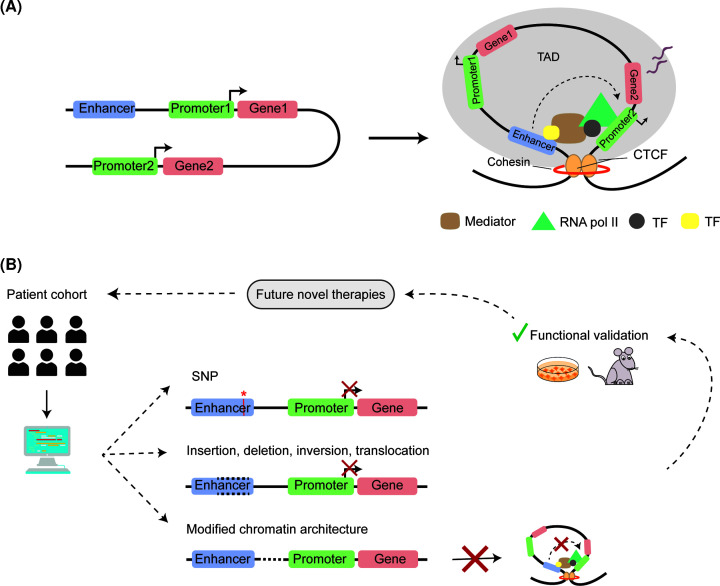
Enhancer–promoter interaction regulates gene expression (**A**) Overview of enhancer–promoter interaction restricted within the TAD. The genome (black line) is organized by domains named TADs, which are established and defined by CTCF insulators and their interaction with the cohesin complex. To establish the enhancer–promoter interaction loop, TFs bind to both enhancer and the target promoter where the RNA Pol II complex is assembled to start transcription of the target gene. Other proteins, such as the Mediator complex, connect enhancer and promoter by interactions with TFs and the transcriptional machinery. (**B**) Flowchart illustrating how genetic variants in NCRE can contribute to disease, and how this knowledge could be utilized to develop future therapies. Through NGS-based studies and other future diagnostic approaches, variants in NCREs are identified. Computational analysis will help to prioritize possibly disease causing variants. These can include SNPs, insertions, deletions, inversions or translocations of enhancers; and alterations in chromatin looping preventing the proper formation of TADs, all potentially influencing or disrupting gene regulatory mechanisms and, therefore, gene expression output. The functional validation of identified variants can help to establish their pathogenicity and can provide cues for the development of future novel therapies targeting NCREs and gene regulatory mechanisms benefitting patients.

Both proximal and distal NCREs interact with target genes to control gene expression, and these include promoters, enhancers, silencers and insulators. Promoters are located next to the transcription start site of a gene, and switch the gene transcription on and off. Spatio-temporal regulation of promoter activity is enabled by the interaction of promoters with other distal NCREs, such as enhancers. Enhancers are relatively short sequences (100–1000 bp) that can reside in exons, introns or intergenic regions, which can be located in close proximity of target genes, but also being distantly located up to megabases (Mb) away from their targets [9]. Enhancers contain multiple binding sites for transcription factors (TFs), which recruit cofactors that together activate transcription of target genes, independently of the enhancer orientation [9,10]. Other predictive features of enhancers include their location in open chromatin when active and epigenetic modifications like enrichment of histone modifications, such as monomethylation of lysine 4 of histone H3 or acetylation of lysine 27 of histone H3 (H3K4me1 and H3K27ac) [11]. Indeed, TF binding capacity to enhancers can be influenced by DNA methylation and chromatin organization, although some TFs also have pioneer factor activity, which means that they can actively open-up closed chromatin regions, enabling activation of genes during development [12,13]. Enhancers are ultimately brought into close proximity to their target promoters by chromatin looping, enabling gene expression [9,14,15]. These enhancer–promoter interactions are mainly confined to topologically associated domains (TADs) and occur in open-chromatin regions. TADs are genomic domains with an average size of ∼1 Mb that encompass regions that preferentially physically interact with other sequences within the same TAD, being delimited and maintained by boundary elements (insulators) [16,17] (Figure 1A). In contrast with enhancers, silencers are NCREs that upon binding of regulatory proteins repress gene expression [18].

There is accumulating evidence that many genes are regulated by more than one enhancer. This enhancer redundancy has been first described in *Drosophila* and is referred to as ‘shadow enhancers’, which drive (partially) overlapping spatio-temporal activity, conferring a level of redundancy and enforcing resistance to genetic variations [19,20]. Recently, the term ‘super enhancer’ (SE) has been coined which describes clusters of putative enhancers in close genomic proximity with unusually high potential to activate transcription of key genes that define cell identity [21–24]. SEs were proposed to differ from putative enhancers in size, TF density and sensitivity to perturbation [21,22,25,26]. Despite this interesting concept, a number of studies have challenged this view and have proposed that SEs are simply a collection of multiple enhancers located in close proximity, that together do not result in more enhancer activity than the sum of its parts [24,27]. Their precise role needs to be further investigated.

### How to identify putative and functional NCREs

To identify putative NCREs, one can take advantage of the several distinct features at different epigenome layers that allow to distinguish NCREs from other non-coding sequences, such as specific chromatin marks, binding of TFs, chromatin accessibility and structural organization, and a variety of techniques that can detect these features.

Chromatin immunoprecipitation (ChIP) was introduced to study protein–DNA interactions, and its combination with NGS allows to determine genome-wide binding profiles of proteins of interest [28,29]. When applied to NCRE-associated proteins, such as lineage-specific TFs or enhancer-associated histone modifications such as H3K4me1 and H3K27ac, it allows to identify the location of putative enhancers genome-wide [29,30]. It is, however, important to note that not all of these putative enhancers will turn out to be ‘*bona fide*’ active enhancers. For example, several studies have shown that not all H3K27ac marked sites function as enhancers in validation experiments, and the genome-wide depletion of some of the enhancer associated marks seem to strikingly impact very little on gene expression [31–34]. This emphasizes that even though ChIP-seq of histone modifications and enhancer-associated TFs is very useful to identify putative enhancers, functional validation remains crucial to confirm these findings [31,35–37].

Another feature of enhancers is their need to be accessible for TFs and structural proteins, and therefore, they reside in open chromatin when active. These accessible DNA regions can be identified by multiple techniques, including the Assay for Transposase Accessible Chromatin with high-throughput sequencing (ATAC-seq). ATAC-seq takes advantage of transposons such as Tn5, which preferentially integrate into open chromatin regions and inserts adapters by tagmentation [38]. These regions are then amplified and sequenced by NGS. One of the main advantages of ATAC-seq is that with minimal input material it can also identify other NCREs, such as promoters, which reside in accessible chromatin as well.

Chromatin architecture plays an essential role in gene regulation, facilitating enhancer–promoter interactions. Several methods exist to assess chromatin conformation. These include, among others, high‐throughput chromosome conformation capture (Hi-C), which allows high-throughput contact mapping of genome-wide interactions, which allows to identify enhancer–promoter interactions and TADs [39]. Other techniques include methods that study the genome-wide interactions of selected regions of interest (such as promoter capture Hi-C [40]) or targets bound by a protein of interest (such as HiChIP [41]). Together, these and other approaches allow to map chromatin conformation interactions, and the knowledge obtained from this can be used to predict the location of putative enhancers.

Although ChIP-seq, ATAC-seq and chromatin conformation assays are able to identify putative NCREs, functional validation remains crucial as not all putative enhancers in fact are real enhancers [42–44], even when localized in open-chromatin regions [33,45]. Classically, this validation was done by low throughput reporter assays, but new technologies enable us to significantly upscale functional validations. These include massively parallel reporter assays (MPRAs), such as STARR-seq [32], that allows the determination of enhancer activity on an episomal plasmid in a high-throughput manner, and Clustered Regularly Interspaced Short Palindromic Repeats (CRISPR)-based screens in which NCREs can be edited in high-throughput at endogenous loci, allowing to directly assess effects on endogenous gene expression [46]. MPRAs were first developed by Patwardhan et al*.* in 2009 for promoter assays and enables the analysis of thousands of regulatory elements in a single experiment [47,48]. Libraries of thousands of enhancers are *en masse* cloned in a reporter construct containing a unique barcode for each enhancer candidate [49]. The RNA-seq readout of these libraries after expression in cells or even *in vivo* in animal models is used to determine the enhancer function by linking the abundance of barcodes in mRNA to the element’s activity [47]. This not only allows the testing of pre-selected genomic regions of choice, but also the screening of randomly fragmented sequences open chromatin regions, regions associated with TFs or histone marks, or even whole genomes [31,32,45,50–52]. Similar approaches can also be used to study the effects of genetic variants on function of putative enhancers via saturation mutagenesis experiments [53]. Together, with all these different currently available tools, the field now seems for the first time ready to start deciphering the regulatory grammar of the non-coding genome, and provide functional annotation to non-coding sequences. Knowledge obtained from this will enable us to better interpret genetic variation encountered in patients and might elucidate unknown causes of disease in the field of human genetics (Figure 1B).

### Non-coding variants in the context of genetic brain disease

The fact that evolution has resulted in such complex, well-organized processes of gene regulation, and the obvious need for these processes for the development of an organism [54], let it not come as a surprise that disturbance of these mechanisms can lead to human disease. Aberrations of NCREs have indeed been implicated in the pathogenesis of human disorders. A classic example is pre-axial polydactyly, where variants in a limb-specific enhancer, the so-called ZRS region located in intron 5 of the *LMBR1* gene, causes disturbed expression of *SHH*, located more than 1 Mb away, resulting in pre-axial polydactyly [55,56]. Other examples include a variant in a silencer of *NOTCH1*, which was shown to contribute to Tetralogy of Fallot (a congenital heart disease) [57] and translocations of the NCREs of *PAX6* that lead to aniridia [56]. Multiple disease examples show that the human brain seems particularly sensitive to gene expression disbalance [4,43,58], and many neurodevelopmental disorders are linked to chromatin modifiers [5,59–64] and architectural proteins with roles in establishing chromatin conformation, such as CTCF, YY1 and STAG1 [1,3,4,65,66]. In this section, we will focus on a number of recent examples that illustrate the wide range of alterations of gene regulatory processes that can cause brain-related disorders, and the novel approaches to identify them. These alterations can range from deletions or duplications of NCREs identified by whole-genome sequencing (WGS) or copy number analysis, point mutations in NCREs detected by WGS or targeted sequencing, disruption of TAD boundaries encountered by chromatin conformation studies, and dysfunction of proteins involved in gene regulatory processes, just to mention a few (for further in-depth review, see [1,43,67,68]) (Figure 1B).

Primary familial brain calcification (PFBC) is a rare microvascular calcifying disorder presenting with neuropsychiatric symptoms, which is caused by haploinsufficiency of an inorganic phosphate transporter, *SLC20A2.* Recently, a deletion encompassing an enhancer region upstream of *SLC20A2* was identified in three patients with PFBC [69], and resulted in decreased *SLC20A2* expression and phosphate uptake to a similar level as observed in *SLC20A2* haploinsufficiency. Similar enhancer deletions have been found in proximity of *GABRD*, a susceptibility factor for juvenile myoclonic epilepsy [70], in a study that performed WGS analysis in an epilepsy cohort [71]. In 28.8% of 198 patients, copy number variants were found near known epilepsy genes, indicating that disruption of the gene regulatory landscape of these genes might be the cause of epilepsy [71]. Two studies have employed targeted sequencing of putative NCREs and provided evidence of single nucleotide alterations at NCREs being causative of developmental disorders. Based on targeted NCRE sequencing of 7930 individuals from the Deciphering Developmental Disorders (DDD) study, it was estimated that 1–3% of patients with neurodevelopmental disorders carry *de novo* pathogenic variant in fetal brain-active NCREs [72], although the number of NCREs investigated in this study was rather limited, possibly underestimating the real contribution of genetic variants in NCREs. Using another targeted sequencing approach, bi-allelic variants in NCREs were found in approximately 5% of consanguineous cases of autism spectrum disorder (ASD) [73]. Interestingly, these variants are located in human accelerated regions (HARs), which are conserved genomic loci with elevated divergence in humans. Won et al*.* further confirmed that non-coding elements in HARs and human gained/lost enhancers interact with genes involved in human brain development, including risk genes for ASD, developmental delay and schizophrenia [74].

One of the challenges in the field remains the linking of encountered non-coding variants from WGS or GWAS to their target genes and, therefore, to neurodevelopmental disorders, as functional annotation of NCREs is still limited. Rapidly accumulating chromatin interaction data may change this. For example, several GWAS risk loci could be assigned to schizophrenia-associated genes based on 3D chromatin interaction maps of developing human cortex derived using Hi-C [75]. Hi-C is even about to enter the clinical setting for individual patients. In a recent study, Hi-C maps of cells derived from nine patients presenting with developmental disorders and harboring structural variants revealed altered TADs and regulatory elements [76]. One of these structural variants from a patient caused altered levels of *FOXG1*. Since variants in *FOXG1* and its regulatory elements were reported in congenital Rett syndrome and Rett syndrome-like phenotypes, the new variant is likely underlying the condition of this individual [77]. Interestingly, TAD-disruption appears to be a common mechanism underlying developmental disorders [78]. Deregulation of gene expression was reported in 7.3% of 219 cases of neurodevelopmental disorders, including ten individuals with structural variants disrupting the regulation of *MEF2C*, which causes intellectual disability (ID), epilepsy and cerebral malformations [79–83]. Patients carrying TAD-disrupting variants distal to *MEF2C* had lower *MEF2C* expression (as assessed in lymphoblastoid cell lines), which suggests TAD disruption as a likely explanation of patient condition.

Another challenging point in variant analysis and functional validation is the tissue-specificity of NCREs (reviewed in [43,84]), and this might be overcome by integrating data derived from relevant *in vitro* models, such as induced pluripotent stem cell (iPSC)-derived or primary neuronal cells. Combination of chromatin accessibility analysis in iPSC-derived excitatory neurons, and risk variants identified in GWAS, allowed the identification of 108 potentially functional loci in NCREs, that might contribute to schizophrenia [85]. A risk variant located at the *MIR137* locus, at a neuron-specific TF-binding site, was confirmed to alter the chromatin accessibility of the *MIR137* promoter. This resulted in altered gene expression which changed dendrite and synapse formation. Another comprehensive study in four iPSC-derived neuronal cell types integrated Hi-C, open chromatin and RNA-seq data to identify long-range enhancer–promoter interactions connecting NCREs to their target genes [86]. The approach was confirmed by CRISPR interference (CRISPRi) targeting cell type-specific regulatory regions of *CDK5RAP3*. *CDK5RAP3* was down-regulated in excitatory neurons when CRISPRi was directed to regions 1 and 2, whereas the effect of interfering with region 3 was only observed in astrocytes and motor neurons. The generated dataset allowed to link 70% of SNPs from GWASs for 11 neuropsychiatric disorders to the promoter-interacting region in one or more cell type. Nott et al*.* identified NCREs using ATAC-seq and ChIP-seq in major brain cell types from cortical brain tissues obtained from six individuals [87]. Risk variants for neurological and psychiatric disorders were then mapped to these regions. The functional effect of a deletion of the microglia-specific *BIN1* enhancer that contained an Alzheimer’s disease-risk variant, was confirmed in microglia but, not in neurons or astrocytes, which were differentiated from the same CRISPR-Cas9 edited human iPSC. Nevertheless, the high-throughput validation of these data remains challenging as each enhancer variant needs to be tested in the respective cell type.

Interestingly, it is becoming clear that enhancers can also predict the pathogenicity of genes that are associated with them [88]. Using machine learning, Wang et al. established an enhancer-domain score (EDS), which takes several parameters into consideration, including the enhancer-domain size, enhancer redundancy, the number of functional nucleotides, TF-binding sites and tissue-specific activity [89]. This revealed that genes regulated by enhancers with a higher EDS are more likely associated with human diseases than genes with a lower score. Also, a higher EDS was linked to genes resilient against genetic disturbance. In other words, functionally important genes might overcome the effects of functionally disturbing genetic variants by using a set of redundant NCREs for their regulation. Furthermore, it was shown that enhancers with a higher EDS regulate genes which are depleted of *cis*-expression quantitative trait loci (*cis*-eQTLs) [89]. *cis-*eQTLs are loci near a gene of interest which partly explain the variation of gene-expression levels [90]. As EDS correlates variations in regulatory regions with phenotypic effects, it could be used as a tool to find the most clinically relevant enhancers and complement other methods which measure the importance of dosage-sensitive genes. This approach integrated with open chromatin data in iPSC-derived neurons revealed potential causative variants of schizophrenia which also influenced chromatin accessibility [91].

In summary, considerable progress has been made in identifying and linking variants in NCREs to their target genes in various cell types in the context of neurological disorders. The integration of these data with functional validation would not only be crucial to enhance our understanding of the functioning of NCREs and their role in neurodevelopment, but also of immense value to improve diagnostics. One could hypothesize that upon functional annotation and validation of more NCREs, it will become feasible to sequence NCREs at high throughput in genetically unexplained patients with neurogenetic disorders as a routine diagnostic approach, or to use the information derived from NCRE studies as a computational filter in WGS analysis, thereby increasing the diagnostic yield in clinical genetics (Figure 1B).

### Modulating enhancer activity for future disease treatment

**Figure 2:**
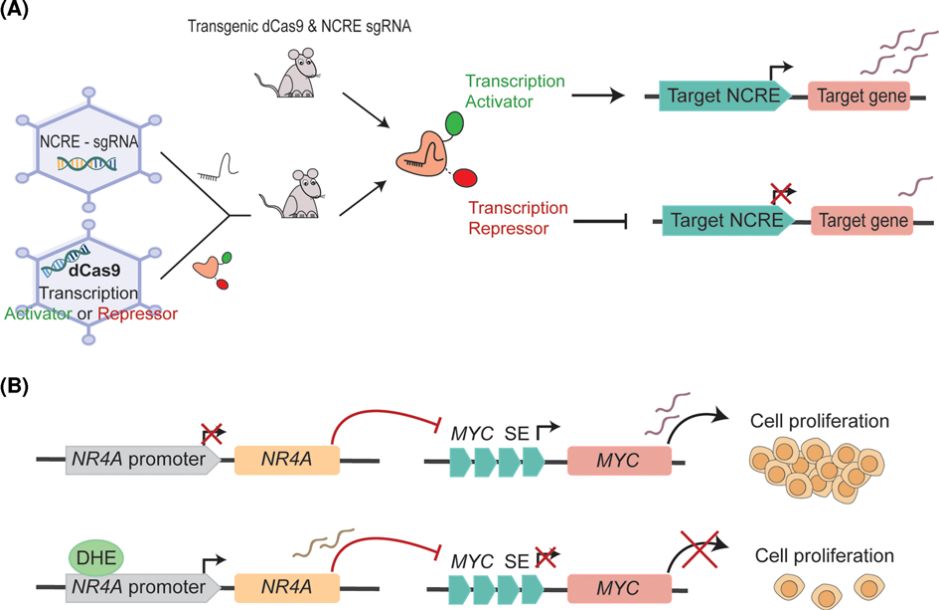
Different approaches to target enhancers as therapeutic agents (**A**) Schematic representation of targeting enhancer activity via dCas9 fused with a transcription activator or repressor to treat disease. Transgenic mice carrying the dCas9 and the single-guide RNA (sgRNA) can either activate or repress the target NCRE and, therefore, promote or down-regulate gene expression, respectively. Both, dCas9 fused with the transcription repressor or activator and sgRNA can also be delivered by two independent recombinant adeno-associated viruses (rAAVs) in mice, which could potentially be translated to the clinic. (**B**) Enhancer activity can be controlled by small molecules like dihydroergotamine (DHE). In absence of DHE, the *NR4A* promoter remains inactive allowing activation of *MYC* SE cluster which results in enhanced *MYC* expression and, consequently, cell proliferation. DHE induces *NR4A* expression by binding to its promoter, and NR4A suppresses the *MYC* SE thus *MYC* expression and cell proliferation in acute myeloid leukemia are inhibited. Similar approaches could be designed for other disorders, based on knowledge obtained from gene regulatory mechanism studies. Abbreviation: dCas9, nuclease-deficient Cas9.

The increasing knowledge of NCREs relevance in neurodevelopmental diseases opens novel strategies for therapeutic interventions. So far, genome-editing strategies to rescue a disease phenotype have mainly focused on protein-coding regions [92]. Although less progress has been made on modifying or influencing NCREs, a number of recent studies show great promise for such ‘enhancer treatment’, either by influencing NCRE activity by chemical compounds or directly altering NCREs using CRISPR-Cas9-based approaches (Figure 2). The examples that we discuss in this section have clearly benefitted from an in-depth functional understanding of NCREs and gene regulatory mechanisms obtained from fundamental studies, reinforcing the need of these to fully benefit translational studies.

An interesting example of the use of small molecules to modify NCRE activity comes from the oncology field. Activation of the oncogene *MYC* is a key driver for acute myeloid leukemia (AML), and is driven by a cluster of enhancers which activity is negatively regulated by the nuclear receptor NR4A [93]. NR4A expression can be induced by dihydroergotamine (DHE), an FDA-approved drug against migraine [94]. This NR4A-inducing activity of DHE was leveraged as an enhancer targeting therapy and resulted in reduced enhancer activity with dismissal of enhancer bound co-activators, reduced H3K27ac and diminished enhancer RNA transcription, ultimately resulting in reduced *MYC* expression and AML cell proliferation [95] (Figure 2B). This illustrates the yet-to-be unraveled potential of small molecules in enhancer treatment, especially when identified in drug repurposing screens, allowing rapid bench to bedside translation.

In the gene therapy field, recombinant adeno-associated virus (rAAV) are promising vectors to re-establish gene expression in genetic disorders to compensate for haploinsufficiency, but this might lead to ectopic transgene expression [96]. CRISPR-mediated gene activation (CRISPRa), in which a nuclease-deficient Cas9 (dCas9) is fused to a transcriptional activator, such as VP64, and is targeted using guide RNAs (gRNAs) to a promoter or enhancer, might overcome these limitations [97]. This approach was recently applied to mice that show obesity due to haploinsufficiency of the genes *SIM* and *Mc4r* [98]. By using gRNAs targeting the hypothalamic enhancer and promoter of *Sim* and the promoter of *Mc4r*, expression of these genes could be up-regulated which was sufficient to rescue the phenotype. As a pre-clinical proof of concept, also rAAV-mediated delivery of CRISPRa was performed demonstrating the possible clinical translation of this approach to humans to treat other gene-dosage abnormalities [98]. A similar approach was applied to a mouse model of Dravet syndrome (DS), a severe epileptic encephalopathy caused by loss-of-function mutations in *SCN1A*. Using rAVV-mediated delivery to the brain of DS mouse pups, dCas9 fused to the transcriptional activator VP160 was targeted by gRNAs to the *Scn1a* promoter and resulted in up-regulated *Scn1a* expression from the wildtype allele [99]. As dCas9 can also be fused to repressive domains [100], similar approaches could also be developed to silence genes, either by directly silencing the promoter or to interfere with other NCREs, and might therefore also provide options to treat disorders caused by gain-of-function mutations [100]. It will be interesting to test whether these approaches could also be further developed to influence gene expression in an allele-specific manner, or to induce expression of specific gene isoforms which are normally not expressed but could rescue phenotypes. The latter could provide a targeted therapy for a severe epileptic encephalopathy caused by a recurrent mutation in *UGP2* (*Barakat–Perenthaler syndrome*, OMIM# 618744), in which the shorter UGP2 isoform is not expressed in brain due to the mutation, and up-regulation of the longer UGP2 isoform which is normally absent from brain could potentially result in a therapy [101].

Obviously, all the approaches discussed in this section will require extensive optimizations and pre-clinical testing to ensure that the right expression levels can be achieved to benefit patients. It is however promising and inspiring for the field, that a first enhancer focused gene therapy that uses CRISPR-Cas9 technology has even recently made it into the clinic [102]. Transfusion-dependent β-thalassemia and sickle cell disease are difficult to treat genetic disorders primary affecting red blood cells, caused by mutations in hemoglobin β [103,104]. Previous work had shown that induction of fetal hemoglobin (HbF) can improve symptoms in both disorders [105]. *BCL11A* is a repressor of γ-globin expression and HbF production in adult erythrocytes, and its expression is under control of the *BCL11A* enhancer. Using CRISPR-Cas9, the *BCL11A* enhancer was deleted in CD34^+^ hematopoietic stem and progenitor cells, enabling autologous stem cell transplantation in β-thalassemia and sickle cell disease patients. This resulted in long-lasting engraftment of the enhancer deleted cells, resulting in increased HbF expression and improved clinical outcomes [102]. This elegantly illustrates how similar approaches could also be designed for other genetic disorders, including those affecting the neurvous system.

## Conclusion

The increasing number of examples where variants in NCREs are linked to human diseases, including neurodevelopmental phenotypes, certainly warrants their evaluation in a clinical diagnostic setting. To further exploit this potential, not only for diagnostics but also for therapies, the field still needs to address many key questions. This includes but is not limited to deciphering the logic behind tissue-specific NCRE activity, the causes and consequences of enhancer redundancy, the establishment of 3D genome organization and the full annotation of all functional non-coding sequences. The many technology advancements in the field, including functional genomics assays and CRISPR-Cas9-based tools, will certainly help to achieve this. But of equal importance will be studying the genetics of human beings, either in large cohorts of healthy individuals to understand normal genetic variation outside of protein-coding genes, or in the context of rare genetic disorders that impact on gene regulatory mechanisms, and which might inform us on new targets and pathways for future therapies. Despite the tremendous potential that NCREs have for therapy, it is clear that it will not be straight forward to develop such treatments easily, as numerous parameters like off-target effects, cell-type specificity, and levels of gene expression need to be defined in order to apply ‘enhancer treatment’ in a wide range of patients. However, the new studies involving enhancer treatment in animal models as well as the first human clinical trials give hope for overcoming the hurdles in the foresight future.

## Summary

- NCREs are important regulators of gene expression.
- Technology innovations and new model systems allow improved functional annotation of NCREs, which increases our capacity to understand genetic variation encountered outside of protein-coding genes.
- There is a rising number of genetic disorders, including neurodevelopmental phenotypes, that is linked to non-coding variants, and the first therapies targeted to NCREs are being developed.
- Increasing our knowledge on NCREs and gene regulatory mechanisms further will enable improved future diagnostics and therapy options for patients with genetic disorders.

## Abbreviations

AML: acute myeloid leukemia
ASD: autism spectrum disorder
ATAC-seq: Assay for Transposase Accessible Chromatin with high-throughput sequencing
ChIP: chromatin immunoprecipitation
*cis*-eQTL: *cis*-expression quantitative trait loci
CRISPR: Clustered Regularly Interspaced Short Palindromic Repeats
CRISPRa: CRISPR-mediated gene activation
CRISPRi: CRISPR interference
dCas9: nuclease-deficient Cas9
DDD: Deciphering Developmental Disorders
DHE: dihydroergotamine
DS: Dravet syndrome
EDS: enhancer-domain score
gRNA: guide RNA
GWAS: genome-wide association study
H3K27ac: acetylation of lysine 27 of histone H3
H3K4me1: monomethylation of lysine 4 of histone H3
HAR: human accelerated region
HbF: fetal hemoglobin
Hi-C: high‐throughput chromosome conformation capture
iPSC: induced pluripotent stem cell
Mb: megabase
MPRA: massively parallel reporter assay
NCRE: non-coding regulatory element
NGS: next-generation sequencing
PFBC: primary familial brain calcification
rAAV: recombinant adeno-associated virus
SE: super enhancer
SNP: single nucleotide polymorphism
TAD: topologically associated domain
TF: transcription factor
WGS: whole-genome sequencing
